# Bridging the Gap: Rewritable Electronics Using Real-Time Light-Induced Dielectrophoresis on Lithium Niobate

**DOI:** 10.1038/s41598-017-09877-9

**Published:** 2017-08-29

**Authors:** Justin R. Sperling, Steven L. Neale, Alasdair W. Clark

**Affiliations:** Biomedical Engineering Research Division, School of Engineering, University of Glasgow, Glasgow, UK

## Abstract

In the context of micro-electronics, the real-time manipulation and placement of components using optics alone promises a route towards increasingly dynamic systems, where the geometry and function of the device is not fixed at the point of fabrication. Here, we demonstrate physically reconfigurable circuitry through light-induced dielectrophoresis on lithium niobate. Using virtual electrodes, patterned by light, to trap, move, and chain individual micro-solder-beads in real-time via dielectrophoresis, we demonstrate rewritable electrical contacts which can make electrical connections between surface-bound components. The completed micro-solder-bead bridges were found to have relatively low resistances that were not solely dominated by the number of interfaces, or the number of discrete beads, in the connection. Significantly, these connections are formed without any melting/fusing of the beads, a key feature of this technique that enables reconfigurability. Requiring only a low-power (~3.5 mW) laser source to activate, and without the need for external power supply or signal generation, the all-optical simplicity of virtual-electrodes may prove significant for the future development of reconfigurable electronic systems.

## Introduction

Using patterned light to create electric-field gradients that impart force onto micro-scale objects forms the basis for ‘touchless’ manipulation techniques such as optical-tweezing (OT) and opto-electronic-tweezing (OET)^1, 2^. Optical systems offer advantages over other current nano and micro-electronic assembly techniques, which either do not allow components to be repositioned after initially being placed^3–6^, are unable to perform highly accurate assembly^4, 7, 8^, require complex and expensive positioning equipment^9–11^, or work only in the assembly of ferroelectronic components^12^. As a result, it has been posited that the incorporation of optical methods into microelectronic device fabrication may provide a path toward the next-generation of low-power manufacturing and assembly tools for the increasingly advanced microelectronic systems of the future.

One such manufacturing concept where the aforementioned attributes of optical manipulation may be particularly suited is that of evolvable hardware; hardware capable of physically transforming based on internal feedback and external input^13, 14^. Capable of real-time, highly-accurate component positioning that does not rely on physical intervention, optical manipulation techniques could provide a pathway to novel examples of dynamically reconfigurable electronic circuitry. Currently, the movement of charge around micro-electronic devices can be dynamically altered using software programmable integrated circuits (ICs). With the vast number of transistors available on modern ICs, this offers almost limitless configurability. However, there is a maximum current value that these transistors can handle, making them unsuitable for switching applications in high-power systems, which typically employ fixed, single-purpose relays. Recent research has shown that it is possible to create reconfigurable electric^15^ and magnetic^16^ circuits on a nanoscale. However, to fully realize the potential of evolvable hardware^13, 14^ in the context of electronics, it will be necessary to construct, move, and reconfigure metallic tracks with micrometer widths over millimeter lengths, such as those used commonly on printed circuit boards. Both OT and OET have drawbacks that may limit their use in such a setting; (1) The force generated by the light (whether directly, in the case of OT, or indirectly, in the case of OET) is only present under direct light illumination^1, 2^ and (2) Miniaturization is problematic: OT relies on high-power lasers to create highly-confined gradient force traps^1^, while the virtual electrodes that underpin OET requires the presence of a top-electrode and a signal generator^2^.

Real-time light patterned dielectrophoresis on the ferroelectric crystal lithium niobate (LN) offers a viable alternative for optical manipulation. LN has been demonstrated as a powerful dielectrophoresis (DEP) tool in the separating, sorting, and patterning of both organic and inorganic nano and micro-particles via both the photorefractive effect^17–22^ and the pyroelectric effect^23–27^. The light-induced DEP forces generated by selectively illuminated LN are due to a combination of both pyroelectric and photorefractive effects^28, 29^.

Here, we demonstrate an application of light-induced DEP on LN as an all-optical manipulation tool for the creation of reconfigurable discrete metallic micro-tracks (45 µm wide). Using a continuous wave 633 nm diode laser at lower-power (3.5 mW) with high-intensity (spot radius of 7 µm, corresponding to an intensity of 2.3 kW·cm^−2^), we create rewritable circuitry using discrete metallic micro-solder beads. Significantly, the fields generated by the electrodes do not disappear when the optical stimulation is removed, as is the case with other optical tweezing systems. This feature allows for the prolonged pinning of particles to certain locations on the surface, and enables particle chaining by positioning new particles beside those pinned to the surface via the previous stimulation. We show that particles are held by this force for several minutes, long enough to construct arrangements of 3–11 particles that are able to conduct electric current while still remaining discrete components.

## Results and Discussion

**Figure 1:**
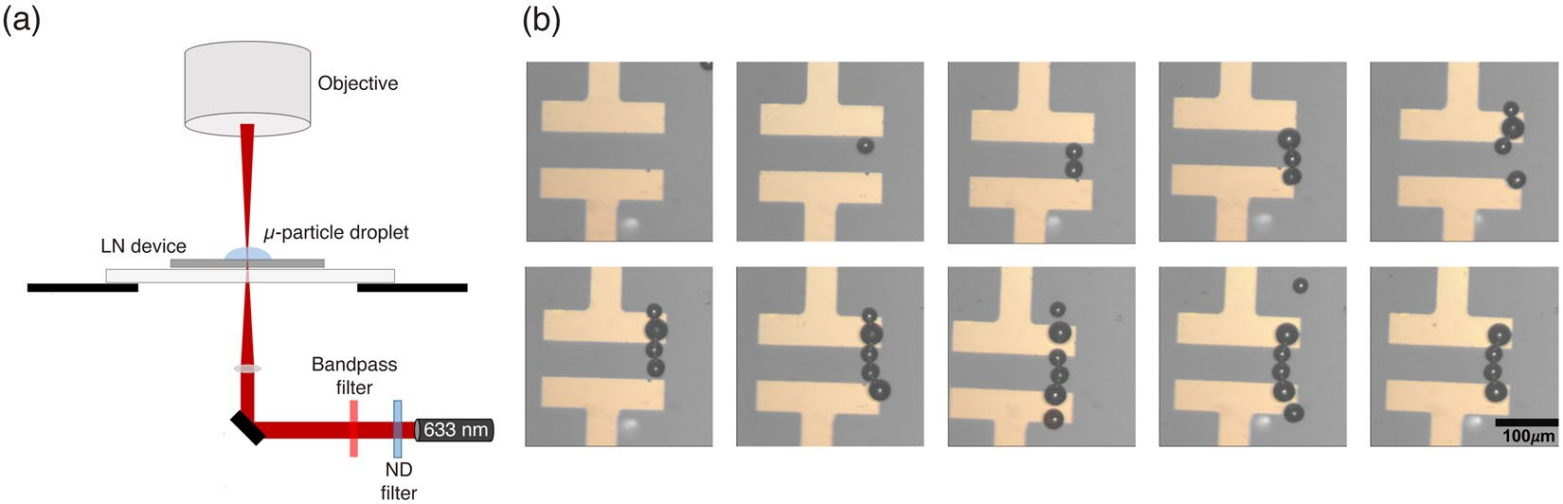
Schematic of the experimental setup, and a demonstration of one-by-one micro-solder-bead assembly between two gold electrodes. (**a**) The schematic demonstrates how the surface of a lithium niobate substrate is activated with a 633 nm continuous wavelength diode laser. This induces a virtual electrode on the top surface of the substrate, which, through dielectrophoresis, is used to trap and manipulate micro-solder-beads. (**b**) Assembly of the micro-solder bead bridge was achieved in approximately eight minutes, where the prolonged trapping mechanism allowed for precise micro-bead placement with a single laser spot.

To demonstrate proof of concept for this technique, it must be established that virtual-electrodes can be used to construct conductive metallic tracks between two permanent electrodes. To this end, lithographically-defined gold electrodes, 60 µm apart, were patterned on an undoped, z-cut LN substrate. Z-cut LN was chosen because, upon stimulation, this crystal orientation produces an electric field that extends in the z-direction and drops off radially in the plane of the substrate, allowing us to create localized electric gradient forces in the exact position of laser stimulation. Metallic micro-solder beads (Sn62Pb36Ag2) with diameters varying from 25–45 µm, suspended in almond oil (selected for its low electro-conductivity and dielectric constant, both favorable for positive DEP), were placed on the surface of the device. When stimulated by a 3.5 mW, 633 nm laser spot (Fig. 1a) a localized inhomogeneous electric field was produced. Using the microscope stage to maneuver this stimulation point, and thus the virtual electrode relative to the substrate, individual micro-solder beads could be trapped, moved, and pinned to the LN surface via positive DEP (Fig. 1b).

Once laser stimulation was removed from a particular point on the substrate, the electric field remained, allowing precise placement of the micro-solder beads, which were chained together, one at a time, between the 60 µm permanent electrode gap (Fig. 1b). To maneuver a bead, the laser spot was placed 5 µm to 10 µm from it, pulling it toward the center of the spot. The spot was then moved in steps of 5 µm to 10 µm, either dragging the bead along with it or causing the bead to jump from spot to spot. Bridge assembly across the 60 µm gap took place in approximately 8 minutes. Using a control substrate composed of glass rather than LN resulted in no bead manipulation taking place, signifying that the mechanism was not a product of optical tweezing. The maximum velocity of the solder beads under the influence of the virtual electrodes was measured to be 151.0 ± 2.6 µm·s^−1^, suggesting that we are generating a force equivalent to 9.98 nN using this method. Assuming that the solder beads are much more polarizable than the oil they are suspended in^2^ we can assign them a Clausius-Mossotti factor of 1, which would then mean that this force comes from a gradient of the electric field squared of 2.4 × 10^15^ V^2^ m^−3^. Significantly, the force is 2.4 times larger than has been reported for micro-solder bead movement using OET^2^, while using a setup that is far simpler to implement. Spectrophotometry analysis of the LN wafer shows an absorption coefficient of 2.83 cm^−1^ at 633 nm, which indicates roughly 14% of the laser light (0.49 mW) is absorbed. This low absorbance leads us to believe that the photorefractive effect is the dominant effect in the generation of the electric field gradient.

**Figure 2:**
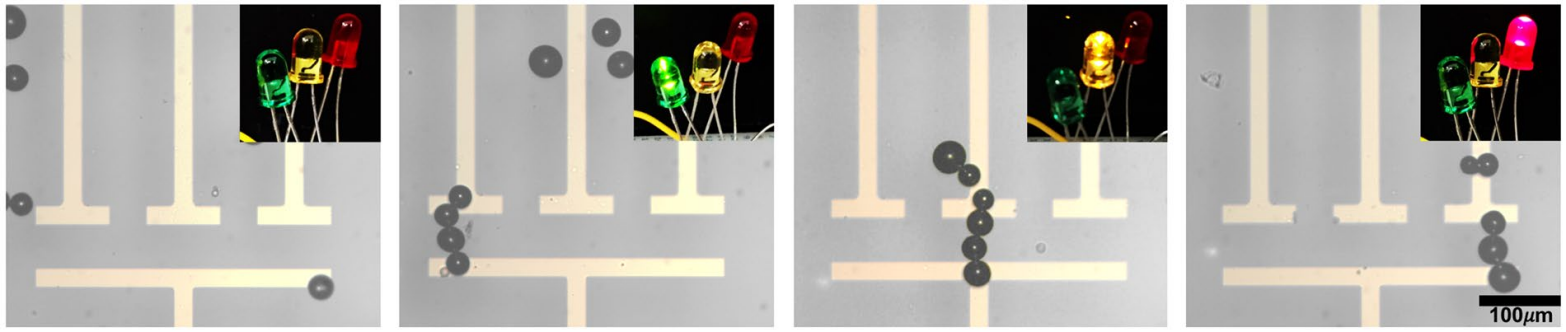
Demonstration of a rewritable three-way switch. The above device consists of a 60 µm gap between a base electrode and three electrodes arranged in parallel to one another on a z-cut LN substrate. Each branch was externally connected to an LED (from left to right: green, amber, and red). The first pane demonstrates that when the electrodes are disconnected, none of the LEDs were on. One-by-one assembly of the micro-solder-beads bridged the gap between the base electrode and one of the top electrodes, completing the circuit and lighting the corresponding LED. After connecting a path to light one LED, a pipette was used to break apart the micro-solder-bead bridge and other nearby micro-solder-beads were used to assemble a new bridge connecting a different path to light a different LED. Each bridge was assembled in around 5 minutes.

Figure 2 demonstrates that the solder-bead bridges produced can not only be used to conduct electricity, but can do so without fusing together, allowing the bridges to be deconstructed and rearranged in real-time to change the flow of electricity around the circuit. A set of permanent electrical paths were fabricated on a LN substrate, each connected to a differently colored LED. A 60 µm gap was included in the design, meaning that in order for any of the circuits to be completed an electrical bridge would have to be constructed across that gap (Fig. 2). Using virtual-electrodes to chain solder beads across these gaps we were able to light each LED in sequence (see Supplementary Video S2). Significantly, each of these connections could subsequently be deconstructed simply by generating fluidic pressure with a pipette, dislodging the beads from their pinned positions. The same beads were then repurposed to complete a different connection to light a different LED.

**Figure 3:**
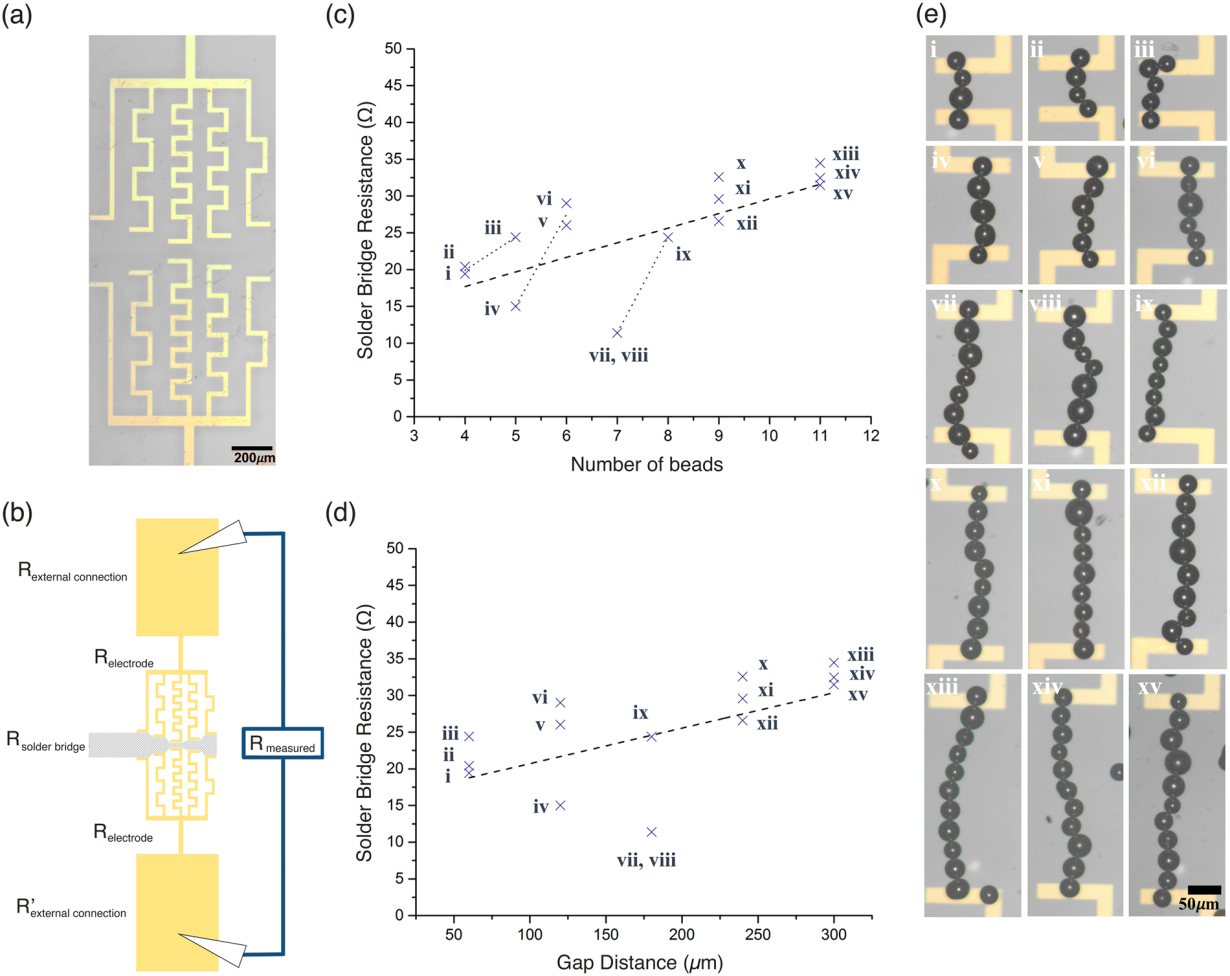
Analysis of solder bridge resistance. (**a**) Image of the device used for building solder bridges between 60 µm, 120 µm, 180 µm, 240 µm, and 300 µm gaps. (**b**) Schematic depicting the source of the resistance terms used in Equation 1 that make up the measured resistance for this device. The measured resistance is a sum of the resistances of both external connections, both electrodes, and the solder bridge itself. To determine scalability of the micro-solder beads as discrete components, chains of beads were assembled between five pairs of electrodes separated by different distances. Upon completing a connection, an ohmmeter connected to the gapped electrodes would register a steady resistance value. The bridge was left constructed for several minutes before deconstruction using fluidic force. Each of the five pairs of electrodes were connected by a solder bridge three separate times, resulting in a total of 15 solder bridge connections, and each newly constructed bridge measured a different resistance value. (**c**) As expected, plotting the measured resistance against the number of beads (or number of interfaces) in each bridge showed a positive correlation. (**d**) Also, plotting the measured resistance against the gap distance covered by the bridge (or the path length of metal in the bridge) also showed a positive correlation. However, it was expected that the number of beads (interfaces) would be the dominating factor in determining the measured resistance as the beads remain discrete. This was not found to be the case. (**e**) Images of each of solder bridges analyzed. The Roman numerals on each image correspond to the points plotted in (**c**) and (**d**).

In order to determine the scalability of these reconfigurable metallic beads as discrete components, their resistance value with increasing chain length was investigated. Figure 3a shows the design of a fixed electrode system that allows us to test the resistance values of particle chains of increasing length. Micro-solder beads were chained between five pairs of electrodes separated by 60 µm, 120 µm, 180 µm, 240 µm, and 300 µm.

Upon completing a connection, the resistance was measured as depicted in the schematic Fig. 3b. By adding the resistances in series, and assuming the resistance of the external connections (∑*R*_*external connections*_, which consists of the probe connections to the metal pads and the leads) to be negligible compared to the other resistances in the circuit, the resistance of each solder bridge connection (*R*_*solder bridge*_) can be approximated by the difference between the externally measured resistance (*R*_*M*_) and the electrode (*R*_*electrode*_), as given by Equation 1.

1 $${R}_{solderbridge} \sim {R}_{M}-2{R}_{electrode}$$

*R*_*M*_ was found to remain constant over several minutes until the micro-solder bridge was deconstructed via fluidic force. For each of the five gapped electrode configurations, three unique bridges utilizing different micro-solder beads were assembled, resulting in a total of 15 micro-solder bridges, as shown in Fig. 3e (i–xv). Each newly constructed bridge measured a different *R*_*M*_, and these values were then used to calculate *R*_*solder bridge*_, which ranged between 11 Ω and 35 Ω (Supplementary Table S1).

As expected, Fig. 3c and d show that an increase to both the number of beads (or interfaces) in the chain and gap distance between electrodes increases the solder bridge resistance. This means that the solder bridge resistance relies on both the number of interfaces in micro-component connections and the length of the chain. The correlation between number of beads in a connection and bridge resistance is further indicated by the dotted lines in Fig. 3c, which correspond to each group of measurements across the same electrode gap (i, ii, iii), (iv, v, vi), and (vii, viii, ix). Each extra bead added on average 2 Ω (Supplementary Figure S3a) and each increment of 60 µm distance added on average 3 Ω (Supplementary Figure S3b).

While a positive correlation between resistance and both the length of the connection and the number of interfaces was expected, what was not expected was the relatively low contact resistance between each micro-solder bead in the connection. With each bead being a discrete component and not melted together, it was expected that the solder bead resistance would be completely dominated by the interfaces between them. However, this was not found to be the case. In fact, it is this relatively low contact resistance that enables longer bead-bridges to remain practically functional as wires, and therefore what makes this reconfigurable system possible.

## Conclusion

In summary, we have demonstrated the use of light-patterned virtual-electrodes to create reconfigurable, conductive metallic tracks between fixed electrical components. Significantly, each metallic bead in these micro-scale chains remains discrete, and does not fuse with its neighbor, allowing full deconstruction and rearrangement of the system in real-time. As a low-power, high-force, all-optical micro-assembly method, the development of this technology may provide a stepping-stone toward the evolvable hardware vision of “Industry 4.0” by providing more circuitry implementations capable of self-repair and dynamic rewiring^13, 14, 30–32^.

## Methods

### Device Fabrication

The devices used in this work were made from double-side mirror polished, 0.5 mm thick, undoped, z-cut lithium niobate purchased from Roditi International Corporation. To lithographically pattern the electrodes, a bi- layer of poly(methyl methacrylate) resist (PMMA) (2.5% 2010, 2.5% 2041) was spun onto the lithium niobate (each layer at 5000 RPM for 60 seconds, followed by a 45 minute bake at 180 °C). 20 nm of Al was then evaporated onto the sample to act as a conduction layer for electron-beam lithography. The resist was patterned using a Vistec VB6 UHR EWF electron beam lithography tool (defining the permanent electrode designs). Microposit Developer CD-26 was then used to remove the electro-conductive aluminum layer. The resist was developed in a 2.5:1 mixture of isopropyl alcohol and methyl isobutyl ketone for 45 seconds at 23 °C. The substrate was cleaned with an O_2_ Plasma Ash at 60 W for 30 seconds. A 2 nm titanium adhesion layer followed by a 25 nm gold layer was then deposited onto the surface using an electron-beam evaporator. Acetone, at 50 °C, was used to lift off the resist, leaving only the electrode pattern on the surface of the substrate. The substrate was epoxy-resin-glued (Mxbon Waterproof Epoxy-E41A) to a glass slide. Electrical wire was bound to each contact point of the gold electrode arrays using conductive silver paint (Agar Scientific, Acheson Silver DAG 1415 M). After the silver paint dried, the electrical wire was further glued using more epoxy resin. These wires were then attached to either an ohmmeter for resistance measurements or an LED-battery-resistor circuit.

### Solder Bead Suspension Preparation

The micro-solder beads used were 62% Sn, 36% Pb, and 2% Ag (Industrie des Poudres Sphériques), with diameters ranging between 25 µm and 45 µm. The solder beads were suspended in almond oil (Almond Oil from *Prunus dulcis*, Sigma-Aldrich).

### Experimental Setup

Figure 1 illustrates the setup of the system. Light from a 25 mW, 633 nm diode laser was directed through an adjustable neutral density filter, which allowed attenuation of the laser power down to 3.5 mW (measured at the substrate). A Nikon 40x microscope objective (N.A. 0.55) was used to focus the laser spot on the device. The solder bead suspension was then placed on the device. The stage was moved to position the laser spot on different regions of the substrate, generating new virtual electrodes, which resulted in solder bead movement. Substrates were viewed using 5x and 10x objectives on a bright-field microscope. All images were recorded using a JAI Color Camera.

### Spectrophotometry Measurements

Transmission and reflection of lithium niobate were measured using a StellarNet Microspectrophotometer.

### Electrode Resistance Measurements

The various micro-gapped arms in Fig. 3a were analyzed using an I/V-sweep from −10 mV to 10 mV with a step size of 0.5 mV on a probe station. Three sweeps were averaged.

### Electrode Path Length/Cross Sectional Area

ImageJ was used to measure the length and width of each section of the micro-gapped arms in Fig. 3a. Metal deposition for all sections of the metal electrodes was performed at the same time and thus assumed to be equal in thickness.

### Data Availability

All data relating to the work outlined in the article can be found here: http://dx.doi.org/10.5525/gla.researchdata.395.

## Electronic supplementary material

Supplementary Information

Supplementary Video S1

Supplementary Video S2
