# Supplementary material for: Bridging the Gap: Rewritable Electronics Using Real-Time Light-Induced Dielectrophoresis on Lithium Niobate
Source: Sci Rep. 2017 Aug 29;7:9660. doi: 10.1038/s41598-017-09877-9 (PMC5575290; doi:10.1038/s41598-017-09877-9)
Supplement: Supplementary file 1 — Supplementary Information [file 41598_2017_9877_MOESM1_ESM.doc]

**Supporting Information**

**Bridging the Gap—Rewritable Electronics with Real-Time Light-Induced Dielectrophoresis on Lithium Niobate**

Justin R. Sperling, Steven L. Neale, and Alasdair W. Clark*

Biomedical Engineering Research Division, School of Engineering, University of Glasgow, UK

**Tweezing Force Analysis**

The maximum force for the tweezers was calculated to be 9.98 nN using Stoke’s Drag Equation with Faxen’s Correction. The dynamic viscosity of almond oil used is 81.92 mPa·s.^[s1]^ The maximum velocity of 151.03 ± 2.6 µm·s^-1^ was measured using video analysis of a solder bead (**Supplementary Video S1**) on the LN stimulated with a continuous wave 633 nm diode laser at 3.5 mW. The bead radius of 13.88 µm was measured from the first frame of the video. For Faxen’s Correction, the height of the center of the solder bead was assumed to roughly equal to the radius of the bead.

**Tweezing Electric Field and Charge Analysis**

The drag force calculated above is equal to the DEP force exerted on the micro-solder bead by the electric field gradient. To calculated the maximum electric field gradient, the equation of a dielectric force for a homogeneous sphere in a dielectric medium was used (**Equation S1)** ^[s2]^


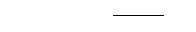
 **(S1)**

where
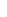
 is the radius of the homogeneous dielectric sphere,
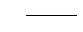
 is the real part of the Clausius-Mossotti (CM) factor with
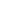
 and
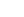
being the complex permittivities of the particle and medium respectively, and
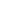
 is the electric field gradient. As the solder is metallic, it is a good conductor and does not permit an internal electric field, thus the CM factor becomes approximately equal to 1. ^[s3]^

Assuming the maximum force exerted on the bead by the tweezing mechanism is when velocity is maximized, the force determined by Stoke’s Drag Equation with Faxen’s Correction from the Tweezing Force Analysis above was plugged into **Equation S1**. Solving for
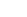
, the maximum electric field gradient was thus 2.4 x 10^15^ V^2^·m^-3^.

**Determining the Absorption Coefficient**

Transmission and reflection of lithium niobate were measured using a StellarNet Microspectrophotometer (**Supplemantary Figure S1**). The absorption coefficient
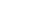
 of the wafer was then determined using **Equation S2** ^[s4]^.


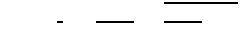
 **(S2)**

where
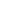
 is the thickness of the wafer and
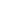
 and
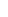
 are the reflectance and transmittance of the wafer at wavelength
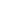
. At 633 nm for a 0.5 mm thick lithium niobate wafer, the measured reflection was 7.95% and transmission was 73.85%, resulting in an absorption coefficient of 2.84 cm^-1^.

**Solder Bead Resistance Analysis**

**Supplementary** **Table S1** contains the measured resistance (*R_M_*) of each connection. The measured resistance (*R_M_*) is the sum of the resistances of the system (*R_solder bridge_, 2R_electrode_,* and *∑R_external connections_*) shown in **Figure 3b**. *∑R_external connections_* is the same for each measured bridge and assumed to be negligible compared to the resistance of *R_solder bridge_* and *R_electrode_*. Therefore

*R_solder bridge_ ~ R_M_ – 2R_electrode_* (S3)

An I/V sweep using micro-probes measured the resistance of the *R_electrode_* region of **Figure 3a** (**Supplementary** **Table S2**). To determine *R_electrode_*, the resistance from the contact between the probe and metal electrode must be subtracted from the measured values of the I/V sweep. The electrodes in the device were metal sheets. Therefore, the resistance of the electrodes follows Pouillet’s Law and is proportional to the path length (*L*) over cross sectional area (*A*). The *L/A* value for each path of the *R_electrode_* region of Figure 3a was calculated using ImageJ measurements (**Supplementary** **Table S3**). The y-intercept of the *L/A* versus the measured sheet resistance curve (**Supplementary** **Figure S2**) is the resistance added by the contact between the probes and the electrode. The contact resistance was subtracted from the measured resistances to determine the resistance of one of the electrode arms in a method similar to a Transmission Line Measurement. Since each arm of the bridged gap is a mirror image of the other, the resulting value was doubled to determine *R_electrodes_* for each gapped electrode path. *R_solder bridge_* was then calculated using **Supplementary** **Equation S3** and plotted versus the number of beads in the bridge between the electrodes (Figure 3a) and the gap distance between electrodes (Figure 3b).

**References**

**[s1]** Fasina, O.O., Hallman, H., Craig-Schmidt, M., Clementsa, C. “Predicting temperature-dependence viscosity of vegetable oils from fatty acid composition.” *J Amer Oil Chem Soc* **83**: 899 (2006).

**[s2]** Grilli, S., Ferraro, P. “Dielectric trapping of suspended particles by selective pyroelectric effect in lithium niobate crystals.” *Appl. Phys. Lett*. **92** (23), 23902 (2008).

**[s3]** Zhang, S., Juvert, J., Cooper, J.M., Neale, S.L., Manipulating and assembling metallic beads with Optoelectronic Tweezers. *Sci. Rep.* **6**, 32840; 10.1038/srep32840 (2016).

**[s4]** Hassanien, A.S., Akl, A.A. “Effect of Se addition on optical and electrical properties of chalcogenide CdSSe thin ﬁlms.” *Superlattices and Microstructures* **89**: 153-169 (2016).


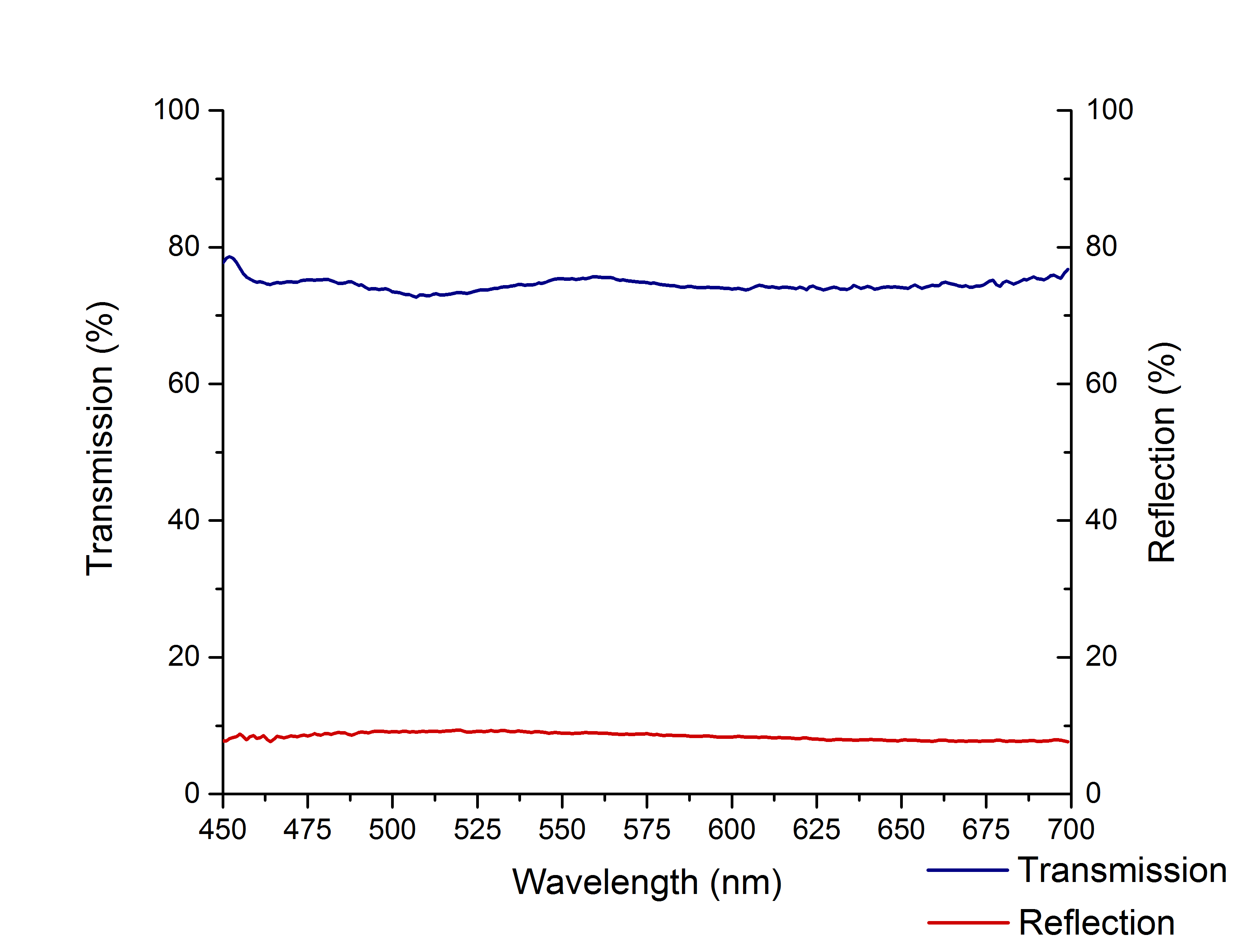


**Supplementary** **Figure S1. Transmission and reflection spectra of LN wafer in the visible spectrum.** At 633 nm for a 0.5 mm thick lithium niobate wafer, the measured reflection was 7.95% and transmission was 73.85%. Using these values in Equation S2, the absorption coefficient at 633 nm is 2.84 cm^-1^.

**Supplementary** **Table S1.** Resistance values for each solder bridge connection made for Figure 3 data set. Measured Resistances from the multimeter were accurate up to 1 Ohm. The Electrode Resistance is calculated using data in **Supplementary** **Table S2** and **Supplementary** **Table S3**. Images of each bridge are in **Figure 3e**.

|  | Arm Gap Distance [µm] | Number of Beads | Measured Resistance [Ω] | Electrode Resistance [Ω] | Solder Bridge Resistance [Ω] |
| --- | --- | --- | --- | --- | --- |
| a. | 60 | 4 | 229 | 209.59 | 19.41 |
| b. | 60 | 4 | 230 | 209.59 | 20.41 |
| c. | 60 | 5 | 234 | 209.59 | 24.41 |
| d. | 120 | 5 | 210 | 194.98 | 15.02 |
| e. | 120 | 6 | 221 | 194.98 | 26.02 |
| f. | 120 | 6 | 224 | 194.98 | 29.02 |
| g. | 180 | 7 | 198 | 186.61 | 11.39 |
| h. | 180 | 7 | 198 | 186.61 | 11.39 |
| i. | 180 | 8 | 211 | 186.61 | 24.39 |
| j. | 240 | 9 | 186 | 159.42 | 26.58 |
| k. | 240 | 9 | 189 | 159.42 | 29.58 |
| l. | 240 | 9 | 192 | 159.42 | 32.58 |
| m. | 300 | 11 | 170 | 138.52 | 31.48 |
| n. | 300 | 11 | 171 | 138.52 | 32.48 |
| o. | 300 | 11 | 173 | 138.52 | 34.48 |

**Supplementary** **Table S2.** The resistance for each of the paths found on the top half of the electrode array seen in **Figure 3a** were measured using a probe station. The *L/A* values are calculated in **Supplementary Table S3**. The resistance of the metal electrodes is proportional to *L/A* by Pouillet’s Law. The y-intercept of Average Measured Path Resistance versus *L/A* (**Supplementary Figure S2**) is a measure of the resistance between the probes and the electrode. In Column 5, the resistance of the probes (21.83 Ω) is removed from the Averaged Measured Path Resistance to obtain the Path Resistance. This value is doubled to produce the Total Electrode Resistance because there are two arms for each electrode array (Column 6).

| 1. Arm Gap Distance [µm] | 2. *L/A* from Supplementary Table S2 [µm^-1^] | 3. Measured Path Resistances [Ω] | 4. Averaged Measured Path Resistance [Ω] | 5. Path Resistance [Ω] | 6. Total Electode Resistance [Ω] |
| --- | --- | --- | --- | --- | --- |
| 60 | 2.85 | 123.46, 128.21, 128.21 | 126.63 | 104.80 | 209.59 |
| 120 | 2.61 | 125.00, 106.38, 126.58 | 119.32 | 97.49 | 194.98 |
| 180 | 2.37 | 120.48, 114.94, 109.89 | 115.10 | 93.30 | 186.61 |
| 240 | 2.14 | 114.94, 97.09, 92.59 | 101.54 | 79.71 | 159.42 |
| 300 | 1.90 | 90.91, 96.15, 86.21 | 91.09 | 69.26 | 138.52 |

Supplementary Table S3. Measured cross sectional area and path length for each *R_electrode_* path of Figure 3b. The Top Section refers to the large, upper portion of the electrode path and the Arm refers to the thinner portion. The lengths of each path of Figure 3a are measured using ImageJ software. Column 8 is a summation of Columns 6 and 7 because the total resistance of resistors in series is their sum.

| 1. Arm Gap Distance [µm] | 2. Top Section Length (*L*) [µm] | 3. Top Section Cross Sectional Area (*A*) [µm^2^] | 4. Arm Length (*L*) [µm] | 5. Arm Cross Sectional Area (*A*) [µm^2^] | 6. Top Section *L/A* [µm^-1^] | 7. Arm *L/A* [µm^-1^] | 8. Total (*L/A*) [µm^-1^] |
| --- | --- | --- | --- | --- | --- | --- | --- |
| 60 | 450 | 1250 | 1556 | 625 | 0.36 | 2.49 | 2.85 |
| 120 | 537 | 1250 | 1364 | 625 | 0.43 | 2.18 | 2.61 |
| 180 | 636 | 1250 | 1164 | 625 | 0.51 | 1.86 | 2.37 |
| 240 | 733 | 1250 | 970 | 625 | 0.59 | 1.55 | 2.14 |
| 300 | 833 | 1250 | 770 | 625 | 0.67 | 1.23 | 1.90 |


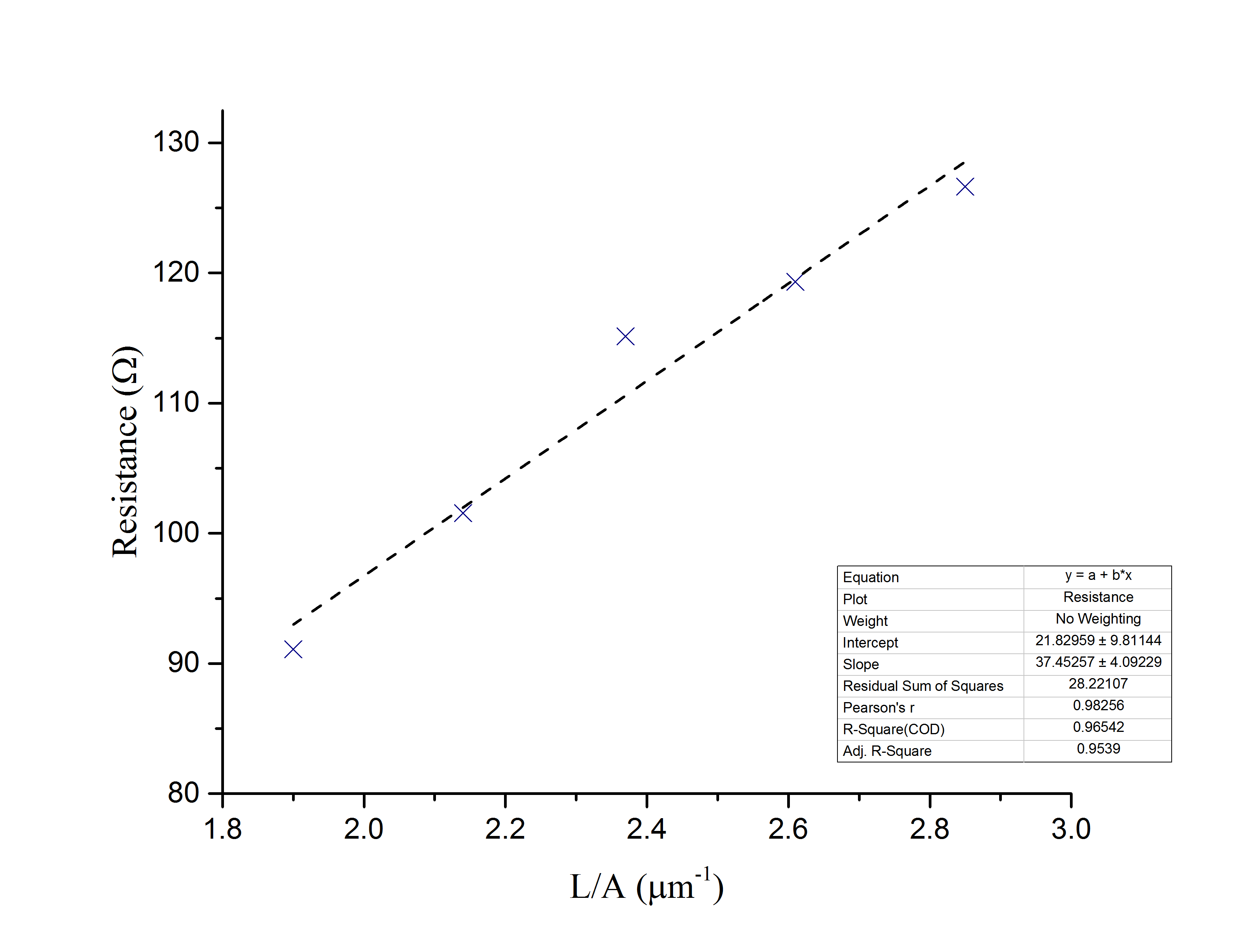


**Supplementary** **Figure S2. Average Measured Path Resistance versus *L/A*.** The resistance of a metal sheet is proportional to *L/A* (Pouillet’s Law). The y-intercept of this plot is 21.83 Ω, which is the resistance between the probes and the metal sheet.


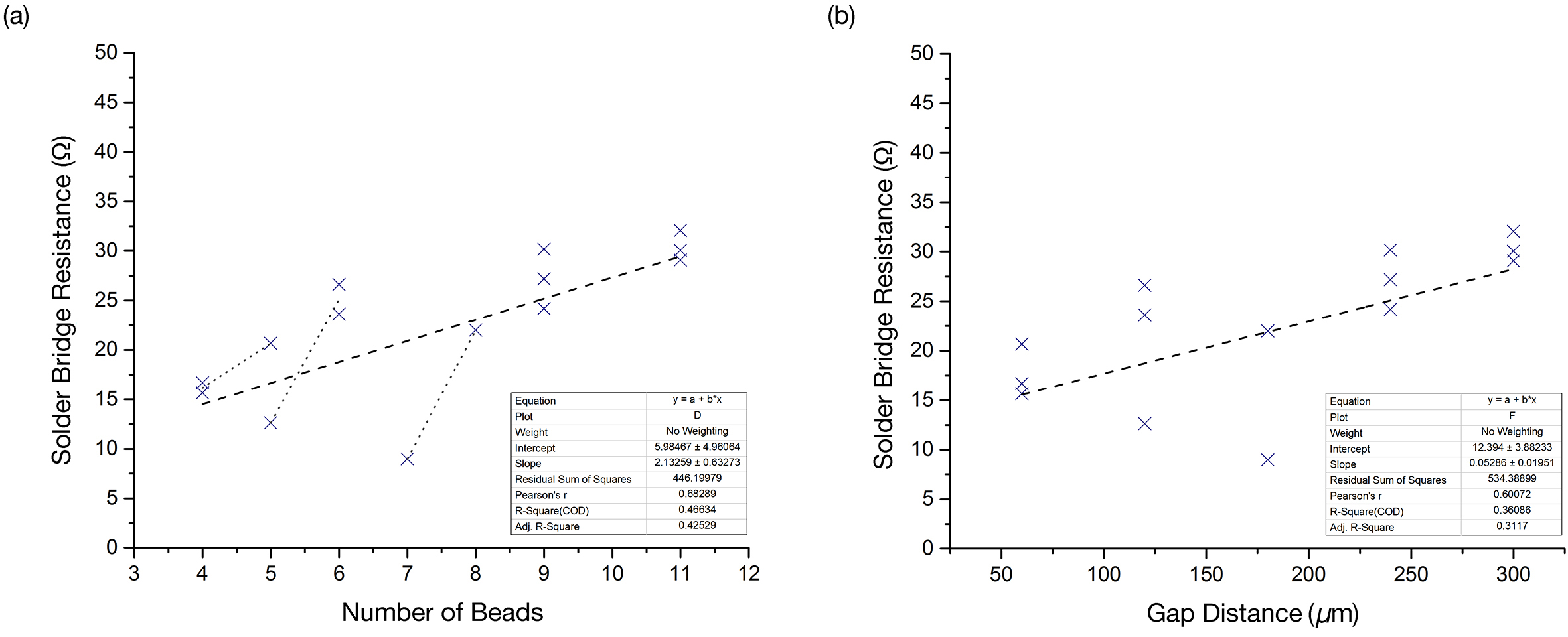


**Supplementary** **Figure S3. Linear fit of solder bridge resistance versus the number of beads between each electrode and gap distance.** The electrical connections created were found to have a resistance between 11 Ω and 35 Ω and this was found to be dependent on both **(a)** the number of beads forming the bridge between the electrodes and **(b)** the distance between electrodes. Each extra bead added on average 2 Ω and each increment of 60 nm distance added on average 3 Ω. The non-zero y-intercept for each plot implies a resistive element to the bridge that does not vary with the number of beads or length of the chain which we ascribe to a different resistance between the beads and lithographically patterned metal electrodes. The low R^2^ values of the graphs show variations in linearity, which may result from the inhomogeneous micro-solder bead diameters from 25 µm to 45 µm (roughly 44% variation).

**Supplementary Video S1.** Video demonstrating the influence of the light-induced DEP field on a single micro-solder bead. This video was used in the Tweezing Force Analysis.

**Supplementary Video S2.** Video demonstrating the light-induced DEP field being used to move micro-solder beads on LN, bridging the gap between two electrodes separated by 60 µm. Once all the interfaces between beads are touching, the LED lights up, indicating the circuit is complete.
